# Supplementary material for: Biostimulant effects of titanium dioxide nanoparticles on germination and initial growth of tomato: evidence of hormesis
Source: PeerJ. 2025 Dec 16;13:e20516. doi: 10.7717/peerj.20516 (PMC12716138; doi:10.7717/peerj.20516)
Supplement: Supplemental Information 2 [file peerj-13-20516-s002.docx]

**Titanium enhances germination, fresh biomass accumulation and initial growth in tomato and stimulates stem and root length in a hormetic manner**

Víctor Hugo Carbajal-Vázquez^1†^, Libia Iris Trejo-Téllez^2†^, Josafhat Salinas-Ruíz^3^ and Fernando Carlos Gómez-Merino^1^*

***Statistical analyses of data of the dependent variables measured to test the effect of titanium on seed weight increase of tomato***

**DATA** WEIGHT GAIN;

INPUT Ti SWIAI;

CARDS;

0.00 141.4035088

0.00 162.6865672

0.00 162.5899281

52.20 184

52.20 135.9649123

52.20 169.5035461

104.40 149.6031746

104.40 182.8685259

104.40 186.9198312

156.60 148.1617647

156.60 168.12749

156.60 145.8515284

208.80 155.6561086

208.80 118.8235294

208.80 128.4552846

ods graphics off;

**PROC** **ANOVA**; CLASS Ti;

MODEL SWIAI=Ti;

MEANS Ti/LSD ALPHA=**0.05**; MEANS Ti; **RUN**;

ANOVA

| **Class level information** | | |
| --- | --- | --- |
| **Class** | **Level** | **Values** |
| **Ti** | 5 | 0 52.2 104.4 156.6 208.8 |
| **No. observations read** | | 15 |
| **No. observations used** | | 15 |

ANOVA

Dependent variable: SWIAI

| **Origin** | **DF** | **Sumof Squares** | **Mean Square** | **F Value** | **Pr > F** |
| --- | --- | --- | --- | --- | --- |
| **Model** | 4 | 2457.148509 | 614.287127 | 1.82 | 0.2024 |
| **Error** | 10 | 3383.069620 | 338.306962 |  |  |
| **Total corr** | 14 | 5840.218129 |  |  |  |

| **R-Square** | **Var Coef.** | **MSE root** | **Mean of SWIAI** |
| --- | --- | --- | --- |
| 0.420729 | 11.78736 | 18.39312 | 156.0410 |

| **Origin** | **DF** | **Anova SS** | **Mean Square** | **F Value** | **Pr > F** |
| --- | --- | --- | --- | --- | --- |
| **Ti** | 4 | 2457.148509 | 614.287127 | 1.82 | 0.2024 |

t Tests (LSD) for SWIAI

| **Alpha** | 0.05 |
| --- | --- |
| **DF** | 10 |
| **Error of Mean Square** | 338.307 |
| **t critical value** | 2.22814 |
| **Least significant difference** | 33.462 |

| **t Groups** | | **Mean** | **N** | **Ti** |
| --- | --- | --- | --- | --- |
|  | A | 173.13 | 3 | 104.4 |
| B | A | 163.16 | 3 | 52.2 |
| B | A | 155.56 | 3 | 0 |
| B | A | 154.05 | 3 | 156.6 |
| B |  | 134.31 | 3 | 208.8 |

| **Ti** | **N** | **SWIAI** | |
| --- | --- | --- | --- |
|  |  | **Mean** | **SD** |
| **0** | **3** | 155.560001 | 12.2599774 |
| **52.2** | **3** | 163.156153 | 24.6385767 |
| **104.4** | **3** | 173.130511 | 20.4757154 |
| **156.6** | **3** | 154.046928 | 12.2487132 |
| **208.8** | **3** | 134.311641 | 19.1018953 |
