# Supplementary material for: Biostimulant effects of titanium dioxide nanoparticles on germination and initial growth of tomato: evidence of hormesis
Source: PeerJ. 2025 Dec 16;13:e20516. doi: 10.7717/peerj.20516 (PMC12716138; doi:10.7717/peerj.20516)
Supplement: Supplemental Information 3 [file peerj-13-20516-s003.docx]

**Titanium enhances germination, fresh biomass accumulation and initial growth in tomato and stimulates stem and root length in a hormetic manner**

Víctor Hugo Carbajal-Vázquez^1†^, Libia Iris Trejo-Téllez^2†^, Josafhat Salinas-Ruíz^3^ and Fernando Carlos Gómez-Merino^1^*

***Statistical analyses of data of the dependent variables measured to test the effect of titanium on germination of tomato seeds***

| **DATA** GERMINATION TITANIUM; | | |  |  |  |  |  |  |  |
| --- | --- | --- | --- | --- | --- | --- | --- | --- | --- |
| INPUT Ti TGP GSC VII VIII RL SL NR NL; | | | |  |  |  |  |  |  |
| CARDS; |  |  |  |  |  |  |  |  |  |
| 0 | 41.403509 | 70 | 0.1842105 | 925.299 | 29.63 | 9.2031429 | 4.0154 | 4.28 | 1.57 |
| 0 | 62.686567 | 100 | 0.1639344 | 1179.09 | 57.51 | 8.5951 | 3.1958 | 3.8 | 1.4 |
| 0 | 62.589928 | 100 | 0.1612903 | 1407.11 | 57.87 | 10.416778 | 3.6543 | 3.55 | 1.33 |
| 52.2 | 84 | 80 | 0.1568627 | 1341.97 | 38.87 | 12.195625 | 4.579 | 3.87 | 1.37 |
| 52.2 | 35.964912 | 90 | 0.1698113 | 1175.17 | 46.75 | 9.4261111 | 3.6313 | 2.88 | 0.77 |
| 52.2 | 69.503546 | 90 | 0.1764706 | 1169.61 | 54.1 | 9.6113333 | 3.3843 | 3.88 | 0.77 |
| 104.4 | 49.603175 | 80 | 0.195122 | 919.68 | 40.04 | 7.5591429 | 3.9368 | 4.14 | 1 |
| 104.4 | 82.868526 | 70 | 0.1320755 | 840.98 | 31.04 | 8.1095 | 3.9045 | 3.66 | 1 |
| 104.4 | 86.919831 | 90 | 0.2045455 | 1068.6 | 38.74 | 8.1555714 | 3.7178 | 3.57 | 0.71 |
| 156.6 | 48.161765 | 90 | 0.1363636 | 1724.6 | 45.89 | 14.363 | 4.7951 | 3.37 | 1 |
| 156.6 | 68.12749 | 100 | 0.2272727 | 1365.1 | 62.72 | 9.9225 | 3.7285 | 2.8 | 1.4 |
| 156.6 | 45.851528 | 80 | 0.1081081 | 1338.32 | 44.39 | 12.618875 | 4.111 | 3.87 | 0.75 |
| 208.8 | 55.656109 | 90 | 0.1607143 | 1460.79 | 46.88 | 12.436125 | 3.7948 | 4 | 1.37 |
| 208.8 | 18.823529 | 100 | 0.1612903 | 1590.83 | 73.39 | 11.7724 | 4.1359 | 3.7 | 1.3 |
| 208.8 | 28.455285 | 100 | 0.1298701 | 1405.27 | 64.86 | 10.136 | 3.9167 | 4.6 | 1.4 |
| ods graphics off; | |  |  |  |  |  |  |  |  |
| **PROC** **ANOVA**; CLASS Ti; | | |  |  |  |  |  |  |  |
| MODEL TGP GSC VII VIII RL SL NR NL=Ti; | | | |  |  |  |  |  |  |
| MEANS Ti/LSD ALPHA=**0.05**; MEANS Ti; **RUN**; | | | |  |  |  |  |  |  |

ANOVA

| **Class level information** | | |
| --- | --- | --- |
| **Class** | **Level** | **Values** |
| **Ti** | 5 | 0 52.2 104.4 156.6 208.8 |

| **No. observations read** | 15 |
| --- | --- |
| **No observations used** | 15 |

Dependent variable: TGP

| **Origin** | **DF** | **Sum of Squares** | **Mean Square** | **F Value** | **Pr > F** |
| --- | --- | --- | --- | --- | --- |
| **Model** | 4 | 2457.148512 | 614.287128 | 1.82 | 0.2024 |
| **Error** | 10 | 3383.069623 | 338.306962 |  |  |
| **Total corr** | 14 | 5840.218135 |  |  |  |

| **R-Square** | **Var Coef.** | **MSE Root** | **Mean for TGP** |
| --- | --- | --- | --- |
| 0.420729 | 32.82081 | 18.39312 | 56.04105 |

| **Origin** | **DF** | **Anova SS** | **Mean Square** | **F Value** | **Pr > F** |
| --- | --- | --- | --- | --- | --- |
| **Ti** | 4 | 2457.148512 | 614.287128 | 1.82 | 0.2024 |

Dependent variable: GSC

| **Origin** | **DF** | **Sum of Squares** | **Mean Square** | **F Value** | **Pr > F** |
| --- | --- | --- | --- | --- | --- |
| **Model** | 4 | 440.000000 | 110.000000 | 0.97 | 0.4651 |
| **Error** | 10 | 1133.333333 | 113.333333 |  |  |
| **Total corr** | 14 | 1573.333333 |  |  |  |

| **R-Square** | **Var Coef.** | **MSE Root** | **Mean of GSC** |
| --- | --- | --- | --- |
| 0.279661 | 12.00656 | 10.64581 | 88.66667 |

| **Origin** | **DF** | **Anova SS** | **Mean Square** | **F Value** | **Pr > F** |
| --- | --- | --- | --- | --- | --- |
| **Ti** | 4 | 440.0000000 | 110.0000000 | 0.97 | 0.4651 |

Dependent variable: VII

| **Origin** | **DF** | **Sum of Squares** | **Mean Square** | **F Value** | **Pr > F** |
| --- | --- | --- | --- | --- | --- |
| **Model** | 4 | 0.00133847 | 0.00033462 | 0.28 | 0.8853 |
| **Error** | 10 | 0.01201915 | 0.00120192 |  |  |
| **Total corr** | 14 | 0.01335762 |  |  |  |

| **R-Square** | **Var Coef.** | **MSE Root** | **MeVII** |
| --- | --- | --- | --- |
| 0.100202 | 21.07139 | 0.034669 | 0.164529 |

| **Origin** | **DF** | **Anova SS** | **Mean Square** | **F Value** | **Pr > F** |
| --- | --- | --- | --- | --- | --- |
| **Ti** | 4 | 0.00133847 | 0.00033462 | 0.28 | 0.8853 |

Dependent variable: VIII

| **Origin** | **DF** | **Sum of Squares** | **Mean Square** | **F Value** | **Pr > F** |
| --- | --- | --- | --- | --- | --- |
| **Model** | 4 | 620924.6811 | 155231.1703 | 5.68 | 0.0119 |
| **Error** | 10 | 273294.2394 | 27329.4239 |  |  |
| **Total corr** | 14 | 894218.9205 |  |  |  |

| **R-Square** | **Var Coef.** | **MSE Root** | **Mean of VIII** |
| --- | --- | --- | --- |
| 0.694377 | 13.11171 | 165.3161 | 1260.828 |

| **Origin** | **DF** | **Anova SS** | **Mean Square** | **F Square** | **Pr > F** |
| --- | --- | --- | --- | --- | --- |
| **Ti** | 4 | 620924.6811 | 155231.1703 | 5.68 | 0.0119 |

Dependent variable: RL

| **Origin** | **DF** | **Sum of Squares** | **Mean Square** | **F Value** | **Pr > F** |
| --- | --- | --- | --- | --- | --- |
| **Model** | 4 | 976.043773 | 244.010943 | 1.93 | 0.1813 |
| **Error** | 10 | 1261.760200 | 126.176020 |  |  |
| **Total corr** | 14 | 2237.803973 |  |  |  |

| **R-Square** | **Var Coef.** | **MSE Root** | **Mean of RL** |
| --- | --- | --- | --- |
| 0.436161 | 22.99669 | 11.23281 | 48.84533 |

| **Origin** | **DF** | **Anova SS** | **Mean Square** | **F Value** | **Pr > F** |
| --- | --- | --- | --- | --- | --- |
| **Ti** | 4 | 976.0437733 | 244.0109433 | 1.93 | 0.1813 |

Dependent variable: SL

| **Origin** | **DF** | **Sum of Squares** | **Mean Square** | **F Value** | **Pr > F** |
| --- | --- | --- | --- | --- | --- |
| **Model** | 4 | 35.10132480 | 8.77533120 | 4.49 | 0.0247 |
| **Error** | 10 | 19.54810588 | 1.95481059 |  |  |
| **Total corr** | 14 | 54.64943069 |  |  |  |

| **R-Square** | **Var Coef.** | **MSE Root** | **Mean of SL** |
| --- | --- | --- | --- |
| 0.642300 | 13.57236 | 1.398145 | 10.30141 |

| **Origin** | **DF** | **Anova SS** | **Mean Square** | **F Value** | **Pr > F** |
| --- | --- | --- | --- | --- | --- |
| **Ti** | 4 | 35.10132480 | 8.77533120 | 4.49 | 0.0247 |

Dependent variable: NR

| **Origin** | **DF** | **Sum of Squares** | **Mean Square** | **F Value** | **Pr > F** |
| --- | --- | --- | --- | --- | --- |
| **Model** | 4 | 0.54085195 | 0.13521299 | 0.75 | 0.5805 |
| **Error** | 10 | 1.80462175 | 0.18046218 |  |  |
| **Total corr** | 14 | 2.34547370 |  |  |  |

| **R-Square** | **Var Coef.** | **MSE Root** | **Mean of NR** |
| --- | --- | --- | --- |
| 0.230594 | 10.89230 | 0.424808 | 3.900080 |

| **Origin** | **DF** | **Anova SS** | **Mean Square** | **F Square** | **Pr > F** |
| --- | --- | --- | --- | --- | --- |
| **Ti** | 4 | 0.54085195 | 0.13521299 | 0.75 | 0.5805 |

Dependent variable: NL

| **Origin** | **DF** | **Sum of Squares** | **Mean Square** | **F Value** | **Pr > F** |
| --- | --- | --- | --- | --- | --- |
| **Model** | 4 | 1.03137333 | 0.25784333 | 1.22 | 0.3625 |
| **Error** | 10 | 2.11640000 | 0.21164000 |  |  |
| **Total corr** | 14 | 3.14777333 |  |  |  |

| **R-Square** | **Var Coef.** | **MSE Root** | **Mean of NL** |
| --- | --- | --- | --- |
| 0.327652 | 12.32920 | 0.460043 | 3.731333 |

| **Origin** | **DF** | **Anova SS** | **Mean Square** | **F Value** | **Pr > F** |
| --- | --- | --- | --- | --- | --- |
| **Ti** | 4 | 1.03137333 | 0.25784333 | 1.22 | 0.3625 |

t Tests (LSD) for TGP

| **Alpha** | | | 0.05 | |
| --- | --- | --- | --- | --- |
| **DF** | | | 10 | |
| **Error of Mean Square** | | | 338.307 | |
| **t critical Value** | | | 2.22814 | |
| **Least significant difference** | | | 33.462 | |
| **Means with the same letter do not have significant differences.** | | | | |
| **t Groups** | | **Mean** | **N** | **Ti** |
|  | A | 73.13 | 3 | 104.4 |
| B | A | 63.16 | 3 | 52.2 |
| B | A | 55.56 | 3 | 0 |
| B | A | 54.05 | 3 | 156.6 |
| B |  | 34.31 | 3 | 208.8 |

t Tests (LSD) for GSC

| Note: | This test controls the Type I comparisonwise error rate, not the experimentwise error rate. |
| --- | --- |

| **Alpha** | | | 0.05 |
| --- | --- | --- | --- |
| **DF** | | | 10 |
| **Error of Mean Square** | | | 113.3333 |
| **t critical Value** | | | 2.22814 |
| **Least significant difference** | | | 19.368 |
| **Means with the same letter do not have significant differences.** | | | |
| **t Groups** | **Mean** | **N** | **Ti** |
| A | 96.667 | 3 | 208.8 |
| A | 90.000 | 3 | 156.6 |
| A | 90.000 | 3 | 0 |
| A | 86.667 | 3 | 52.2 |
| A | 80.000 | 3 | 104.4 |

t Tests (LSD) for VII

| Note: | This test controls the Type I comparisonwise error rate, not the experimentwise error rate. |
| --- | --- |

| **Alpha** | 0.05 |
| --- | --- |
| **DF** | 10 |
| **Error of the Mean Square** | 0.001202 |
| **t critical Value** | 2.22814 |
| **Least significant difference** | 0.0631 |

| **Means with the same letter do not have significant differences.** | | | |
| --- | --- | --- | --- |
| **t Groups** | **Mean** | **N** | **Ti** |
| A | 0.17725 | 3 | 104.4 |
| A | 0.16981 | 3 | 0 |
| A | 0.16771 | 3 | 52.2 |
| A | 0.15725 | 3 | 156.6 |
| A | 0.15062 | 3 | 208.8 |

t Tests (LSD) for VIII

| **Alpha** | 0.05 |
| --- | --- |
| **DF** | 10 |
| **Error of Mean Square** | 27329.42 |
| **t critical Value** | 2.22814 |
| **Least significant difference** | 300.75 |

| **Means with the same letter do not have significant differences.** | | | | |
| --- | --- | --- | --- | --- |
| **t Groups** | | **Mean** | **N** | **Ti** |
|  | A | 1485.6 | 3 | 208.8 |
|  | A | 1476.0 | 3 | 156.6 |
| B | A | 1228.9 | 3 | 52.2 |
| B |  | 1170.5 | 3 | 0 |
| B |  | 943.1 | 3 | 104.4 |

t Tests (LSD) for RL

| Note: | This test controls the Type I comparisonwise error rate, not the experimentwise error rate. |
| --- | --- |

| **Alpha** | 0.05 |
| --- | --- |
| **DF** | 10 |
| **Error of Mean Square** | 126.176 |
| **t critical Value** | 2.22814 |
| **Least significant difference** | 20.435 |

| **Means with the same letter do not have significant differences.** | | | | |
| --- | --- | --- | --- | --- |
| **t Groups** | | **Mean** | **N** | **Ti** |
|  | A | 61.710 | 3 | 208.8 |
| B | A | 51.000 | 3 | 156.6 |
| B | A | 48.337 | 3 | 0 |
| B | A | 46.573 | 3 | 52.2 |
| B |  | 36.607 | 3 | 104.4 |

t Tests (LSD) for SL

| Note: | This test controls the Type I comparisonwise error rate, not the experimentwise error rate. |
| --- | --- |

| **Alpha** | 0.05 |
| --- | --- |
| **DF** | 10 |
| **Error of Mean Square** | 1.954811 |
| **t critical Value** | 2.22814 |
| **Least significant difference** | 2.5436 |

| **Means with the same letter do not have significant differences.** | | | | | |
| --- | --- | --- | --- | --- | --- |
| **t Groups** | | | **Mean** | **N** | **Ti** |
|  | A |  | 12.301 | 3 | 156.6 |
| B | A |  | 11.448 | 3 | 208.8 |
| B | A | C | 10.411 | 3 | 52.2 |
| B |  | C | 9.405 | 3 | 0 |
|  |  | C | 7.941 | 3 | 104.4 |

t Tests (LSD) for NR

| Note: | This test controls the Type I comparisonwise error rate, not the experimentwise error rate. |
| --- | --- |

| **Alpha** | 0.05 |
| --- | --- |
| **DF** | 10 |
| **Error of Mean Square** | 0.180462 |
| **t critical Value** | 2.22814 |
| **Least significant difference** | 0.7728 |

| **Means with the same letter do not have significant differences.** | | | |
| --- | --- | --- | --- |
| **t Groups** | **Mean** | **N** | **Ti** |
| A | 4.2115 | 3 | 156.6 |
| A | 3.9491 | 3 | 208.8 |
| A | 3.8649 | 3 | 52.2 |
| A | 3.8530 | 3 | 104.4 |
| A | 3.6218 | 3 | 0 |

t Tests (LSD) for NL

| Note: | This test controls the Type I comparisonwise error rate, not the experimentwise error rate. |
| --- | --- |

| **Alpha** | 0.05 |
| --- | --- |
| **DF** | 10 |
| **Error of Mean Square** | 0.21164 |
| **t critical Value** | 2.22814 |
| **Least significant difference** | 0.8369 |

| **Means with the same letter do not have significant differences.** | | | |
| --- | --- | --- | --- |
| **t Groups** | **Mean** | **N** | **Ti** |
| A | 4.1000 | 3 | 208.8 |
| A | 3.8767 | 3 | 0 |
| A | 3.7900 | 3 | 104.4 |
| A | 3.5433 | 3 | 52.2 |
| A | 3.3467 | 3 | 156.6 |

| **Ti** | **N** | **TGP** | | **GSC** | | **VII** | | **VIII** | |
| --- | --- | --- | --- | --- | --- | --- | --- | --- | --- |
|  |  | **Mean** | **SD** | **Mean** | **SD** | **Mean** | **SD** | **Mean** | **SD** |
| **0** | **3** | 55.5600013 | 12.2599774 | 90.0000000 | 17.3205081 | 0.16981176 | 0.01253959 | 1170.49967 | 241.020342 |
| **52.2** | **3** | 63.1561528 | 24.6385768 | 86.6666667 | 5.7735027 | 0.16771488 | 0.00997061 | 1228.91667 | 97.946519 |
| **104.4** | **3** | 73.1305106 | 20.4757154 | 80.0000000 | 10.0000000 | 0.17724763 | 0.03940296 | 943.08667 | 115.601125 |
| **156.6** | **3** | 54.0469277 | 12.2487132 | 90.0000000 | 10.0000000 | 0.15724816 | 0.06226696 | 1476.00667 | 215.704140 |
| **208.8** | **3** | 34.3116409 | 19.1018953 | 96.6666667 | 5.7735027 | 0.15062491 | 0.01797648 | 1485.63000 | 95.241260 |

| **Ti** | **N** | **RL** | | **SL** | | **NR** | | **NL** | |
| --- | --- | --- | --- | --- | --- | --- | --- | --- | --- |
|  |  | **Mean** | **SD** | **Mean** | **SD** | **Mean** | **SD** | **Mean** | **SD** |
| **0** | **3** | 48.3366667 | 16.2014485 | 9.4050069 | 0.92746392 | 3.62183333 | 0.41076344 | 3.87666667 | 0.37098967 |
| **52.2** | **3** | 46.5733333 | 7.6165368 | 10.4110231 | 1.54828281 | 3.86486667 | 0.63066795 | 3.54333333 | 0.57448528 |
| **104.4** | **3** | 36.6066667 | 4.8644972 | 7.9414048 | 0.33184901 | 3.85303333 | 0.11822378 | 3.79000000 | 0.30643107 |
| **156.6** | **3** | 51.0000000 | 10.1774899 | 12.3014583 | 2.23720251 | 4.21153333 | 0.54036016 | 3.34666667 | 0.53538148 |
| **208.8** | **3** | 61.7100000 | 13.5328083 | 11.4481750 | 1.18384338 | 3.94913333 | 0.17284746 | 4.10000000 | 0.45825757 |
