# Supplementary material for: Biostimulant effects of titanium dioxide nanoparticles on germination and initial growth of tomato: evidence of hormesis
Source: PeerJ. 2025 Dec 16;13:e20516. doi: 10.7717/peerj.20516 (PMC12716138; doi:10.7717/peerj.20516)
Supplement: Supplemental Information 4 [file peerj-13-20516-s004.docx]

**Titanium enhances germination, fresh biomass accumulation and initial growth in tomato and stimulates stem and root length in a hormetic manner**

Víctor Hugo Carbajal-Vázquez^1†^, Libia Iris Trejo-Téllez^2†^, Josafhat Salinas-Ruíz^3^ and Fernando Carlos Gómez-Merino^1^*

***Statistical analyses of data of the dependent variables measured to test the effect of titanium on water content increase of tomato seeds***

**Data in percentage**

**DATA** WATER CONTENT;

INPUT Ti CHR CHT CHH;

CARDS;

0.00 88.6883273 95.4939341 91.9210054

0.00 92.2340426 95.0985346 91.5433404

0.00 93.5016112 95.3679876 91.4732593

52.20 93.5774947 95.2482270 91.5654952

52.20 92.5449871 95.1456311 91.7888563

52.20 93.5235507 95.1162791 92.1305182

104.40 94.6897708 96.9965870 94.2351598

104.40 91.0630841 95.0354610 92.2110553

104.40 94.2988204 96.1897356 92.2973878

156.60 94.0213971 96.3868816 92.6358854

156.60 93.8851913 95.5248619 91.9825073

156.60 94.4186047 95.7493188 92.4114671

208.80 94.2167322 96.1970075 93.0921053

208.80 95.1171231 95.7598857 93.2548665

208.80 92.8666964 95.1958225 95.7903780

ods graphics off;

**PROC** **ANOVA**; CLASS Ti;

MODEL CHR CHT CHH=Ti;

MEANS Ti/LSD ALPHA=**0.05**; MEANS Ti; **RUN**;

ANOVA

| **Class Level Information** | | |
| --- | --- | --- |
| **Class** | **Levels** | **Values** |
| **Ti** | 5 | 0 52.2 104.4 156.6 208.8 |

| **No. observations read** | 15 |
| --- | --- |
| **No. observations used** | 15 |

Dependent variable: CHR

| **Origin** | **DF** | **Sum of Squares** | **Mean Square** | **F Value** | **Pr > F** |
| --- | --- | --- | --- | --- | --- |
| **Model** | 4 | 13.70100457 | 3.42525114 | 1.44 | 0.2905 |
| **Error** | 10 | 23.76912074 | 2.37691207 |  |  |
| **Total corr** | 14 | 37.47012531 |  |  |  |

| **R-Square** | **Var Coef.** | **MSE Root** | **Mean of CHR** |
| --- | --- | --- | --- |
| 0.365651 | 1.653444 | 1.541724 | 93.24316 |

| **Origin** | **DF** | **Anova SS** | **Mean Square** | **F Value** | **Pr > F** |
| --- | --- | --- | --- | --- | --- |
| **Ti** | 4 | 13.70100457 | 3.42525114 | 1.44 | 0.2905 |

| SAS |
| --- |

ANOVA

Dependent Variable: CHT

| **Origin** | **DF** | **Sum of Squares** | **Mean Square** | **F Value** | **Pr > F** |
| --- | --- | --- | --- | --- | --- |
| **Model** | 4 | 1.73488211 | 0.43372053 | 1.48 | 0.2807 |
| **Error** | 10 | 2.93817983 | 0.29381798 |  |  |
| **Total corr** | 14 | 4.67306194 |  |  |  |

| **R-Square** | **Var Coef.** | **MSE Root** | **Mean of CHT** |
| --- | --- | --- | --- |
| 0.371252 | 0.566798 | 0.542050 | 95.63374 |

| **Origin** | **DF** | **Anova SS** | **Mean Square** | **F Value** | **Pr > F** |
| --- | --- | --- | --- | --- | --- |
| **Ti** | 4 | 1.73488211 | 0.43372053 | 1.48 | 0.2807 |

| SAS |
| --- |

ANOVA

Dependent Variable: CHH

| **Origin** | **DF** | **Sum of Squares** | **Mean Square** | **F Value** | **Pr > F** |
| --- | --- | --- | --- | --- | --- |
| **Model** | 4 | 11.25344578 | 2.81336145 | 3.66 | 0.0439 |
| **Error** | 10 | 7.69685461 | 0.76968546 |  |  |
| **Total corr** | 14 | 18.95030039 |  |  |  |

| **R-Square** | **Var Coef.** | **MSE Root** | **Mean of CHH** |
| --- | --- | --- | --- |
| 0.593840 | 0.947882 | 0.877317 | 92.55555 |

| **Origin** | **DF** | **Anova SS** | **Mean Square** | **F Value** | **Pr > F** |
| --- | --- | --- | --- | --- | --- |
| **Ti** | 4 | 11.25344578 | 2.81336145 | 3.66 | 0.0439 |

| SAS |
| --- |

ANOVA

t Tests (LSD) for CHR

| Note: | This test controls the Type I comparisonwise error rate, not the experimentwise error rate. |
| --- | --- |

| **Alpha** | 0.05 |
| --- | --- |
| **DF** | 10 |
| **Error of Mean Square** | 2.376912 |
| **t critical value** | 2.22814 |
| **Least significant difference** | 2.8048 |

| **Means with the same letter do not have significant differences.** | | | |
| --- | --- | --- | --- |
| **t Group** | **Mean** | **N** | **Ti** |
| A | 94.108 | 3 | 156.6 |
| A |  |  |  |
| A | 94.067 | 3 | 208.8 |
| A |  |  |  |
| A | 93.351 | 3 | 104.4 |
| A |  |  |  |
| A | 93.215 | 3 | 52.2 |
| A |  |  |  |
| A | 91.475 | 3 | 0 |

| SAS |
| --- |

ANOVA

t Tests (LSD) for CHT

| Note: | This test controls the Type I comparisonwise error rate, not the experimentwise error rate. |
| --- | --- |

| **Alpha** | 0.05 |
| --- | --- |
| **DF** | 10 |
| **Error of Mean Square** | 0.293818 |
| **t critical Value** | 2.22814 |
| **Least significant difference** | 0.9861 |

| **Means with the same letter do not have significant differences.** | | | |
| --- | --- | --- | --- |
| **t Groups** | **Mean** | **N** | **Ti** |
| A | 96.0739 | 3 | 104.4 |
| A |  |  |  |
| A | 95.8870 | 3 | 156.6 |
| A |  |  |  |
| A | 95.7176 | 3 | 208.8 |
| A |  |  |  |
| A | 95.3202 | 3 | 0 |
| A |  |  |  |
| A | 95.1700 | 3 | 52.2 |

| SAS |
| --- |

ANOVA

t Tests (LSD) for CHH

| Note: | This test controls the Type I comparisonwise error rate, not the experimentwise error rate. |
| --- | --- |

| **Alpha** | 0.05 |
| --- | --- |
| **DF** | 10 |
| **Error of Mean Square** | 0.769685 |
| **t critical value** | 2.22814 |
| **Least significant difference** | 1.5961 |

| **Means with the same letter do not have significant differences.** | | | | |
| --- | --- | --- | --- | --- |
| **t Groups** | | **Mean** | **N** | **Ti** |
|  | A | 94.0458 | 3 | 208.8 |
|  | A |  |  |  |
| B | A | 92.9145 | 3 | 104.4 |
| B |  |  |  |  |
| B |  | 92.3433 | 3 | 156.6 |
| B |  |  |  |  |
| B |  | 91.8283 | 3 | 52.2 |
| B |  |  |  |  |
| B |  | 91.6459 | 3 | 0 |

| SAS |
| --- |

ANOVA

| **Ti** | **N** | **CHR** | | **CHT** | | **CHH** | |
| --- | --- | --- | --- | --- | --- | --- | --- |
|  |  | **Mean** | **SD** | **Mean** | **SD** | **Mean** | **SD** |
| **0** | **3** | 91.4746604 | 2.49487906 | 95.3201521 | 0.20199348 | 91.6458684 | 0.24083839 |
| **52.2** | **3** | 93.2153442 | 0.58117247 | 95.1700457 | 0.06927927 | 91.8282899 | 0.28456810 |
| **104.4** | **3** | 93.3505584 | 1.99063173 | 96.0739279 | 0.98567863 | 92.9145343 | 1.14450955 |
| **156.6** | **3** | 94.1083977 | 0.27714489 | 95.8870208 | 0.44720338 | 92.3432866 | 0.33198218 |
| **208.8** | **3** | 94.0668506 | 1.13267536 | 95.7175719 | 0.50193196 | 94.0457833 | 1.51305350 |
