# Supplementary material for: Biostimulant effects of titanium dioxide nanoparticles on germination and initial growth of tomato: evidence of hormesis
Source: PeerJ. 2025 Dec 16;13:e20516. doi: 10.7717/peerj.20516 (PMC12716138; doi:10.7717/peerj.20516)
Supplement: Supplemental Information 5 [file peerj-13-20516-s005.docx]

**Titanium enhances germination, fresh biomass accumulation and initial growth in tomato and stimulates stem and root length in a hormetic manner**

Víctor Hugo Carbajal-Vázquez^1†^, Libia Iris Trejo-Téllez^2†^, Josafhat Salinas-Ruíz^3^ and Fernando Carlos Gómez-Merino^1^*

***Statistical analyses of data of the dependent variables measured to test the effect of titanium on production of tomato seedlings***

**DATA** GERMINATION TITANIUM BIOMass;

INPUT Ti PFR PSR PFT PST PFH PSH PFTotal PSTotal;

CARDS;

0.00 0.0831 0.0094 0.1731 0.0078 0.1671 0.0135 0.4233 0.0307

0.00 0.188 0.0146 0.1979 0.0097 0.1892 0.016 0.5751 0.0403

0.00 0.1862 0.0121 0.1943 0.009 0.1982 0.0169 0.5787 0.038

52.20 0.1884 0.0121 0.141 0.0067 0.1565 0.0132 0.4859 0.032

52.20 0.1945 0.0145 0.1545 0.0075 0.1705 0.014 0.5195 0.036

52.20 0.2208 0.0143 0.1720 0.0084 0.2084 0.0164 0.6012 0.0391

104.40 0.1789 0.0095 0.1465 0.0044 0.1752 0.0101 0.5006 0.024

104.40 0.1712 0.0153 0.1128 0.0056 0.1592 0.0124 0.4432 0.0333

104.40 0.1526 0.0087 0.1286 0.0049 0.1493 0.0115 0.4305 0.0251

156.60 0.1589 0.0095 0.1799 0.0065 0.1711 0.0126 0.5099 0.0286

156.60 0.2404 0.0147 0.1810 0.0081 0.2058 0.0165 0.6272 0.0393

156.60 0.1935 0.0108 0.1835 0.0078 0.1779 0.0135 0.5549 0.0321

208.80 0.1781 0.0103 0.1604 0.0061 0.1824 0.0126 0.5209 0.029

208.80 0.3031 0.0148 0.2099 0.0089 0.2209 0.0149 0.7339 0.0386

208.80 0.2243 0.016 0.1915 0.0092 0.2328 0.0098 0.6486 0.035

ods graphics off;

**PROC** **ANOVA**; CLASS Ti;

MODEL PFR PSR PFT PST PFH PSH PFTotal PSTotal=Ti;

MEANS Ti/LSD ALPHA=**0.05**; MEANS Ti; **RUN**;

| SAS |
| --- |

ANOVA

| **Class level information** | | |
| --- | --- | --- |
| **Class** | **Level** | **Value** |
| **Ti** | 5 | 0 52.2 104.4 156.6 208.8 |

| **N.º observations read** | 15 |
| --- | --- |
| **No. observations used** | 15 |

| SAS |
| --- |

ANOVA

Dependent variable: Root Fresh Weight

| **Origin** | **DF** | **Sum of squares** | **Mean square** | **F Value** | **Pr > F** |
| --- | --- | --- | --- | --- | --- |
| **Model** | 4 | 0.01240585 | 0.00310146 | 1.59 | 0.2512 |
| **Error** | 10 | 0.01950679 | 0.00195068 |  |  |
| **Total corrected** | 14 | 0.03191264 |  |  |  |

| **R-square** | **Var Coef.** | **MSE Root** | **Mean** |
| --- | --- | --- | --- |
| 0.388744 | 23.14805 | 0.044166 | 0.190800 |

| **Origin** | **DF** | **Anova SS** | **Square Mean** | **F Value** | **Pr > F** |
| --- | --- | --- | --- | --- | --- |
| **Ti** | 4 | 0.01240585 | 0.00310146 | 1.59 | 0.2512 |

| SAS |
| --- |

ANOVA

Dependent variable: Root Dry Weight

| **Origin** | **DF** | **Sum of squares** | **Mean Square** | **F Value** | **Pr > F** |
| --- | --- | --- | --- | --- | --- |
| **Model** | 4 | 0.00001619 | 0.00000405 | 0.53 | 0.7138 |
| **Error** | 10 | 0.00007573 | 0.00000757 |  |  |
| **Total corr.** | 14 | 0.00009192 |  |  |  |

| **R-square** | **Var Coef.** | **MSE Root** | **Mean** |
| --- | --- | --- | --- |
| 0.176132 | 22.12096 | 0.002752 | 0.012440 |

| **Origin** | **DF** | **Anova SS** | **Mean Square** | **F Value** | **Pr > F** |
| --- | --- | --- | --- | --- | --- |
| **Ti** | 4 | 0.00001619 | 0.00000405 | 0.53 | 0.7138 |

| SAS |
| --- |

ANOVA

Dependent: Shoot Fresh Weight

| **Origin** | **DF** | **Sum of squares** | **Mean Square** | **F Value** | **Pr > F** |
| --- | --- | --- | --- | --- | --- |
| **Model** | 4 | 0.00784421 | 0.00196105 | 7.35 | 0.0050 |
| **Error** | 10 | 0.00266971 | 0.00026697 |  |  |
| **Total corr** | 14 | 0.01051392 |  |  |  |

| **R-square** | **Var Coef.** | **MSE Root** | **Mean** |
| --- | --- | --- | --- |
| 0.746079 | 9.699179 | 0.016339 | 0.168460 |

| **Origin** | **DF** | **Anova SS** | **Mean Square** | **F value** | **Pr > F** |
| --- | --- | --- | --- | --- | --- |
| **Ti** | 4 | 0.00784421 | 0.00196105 | 7.35 | 0.0050 |

| SAS |
| --- |

ANOVA

Dependent variable: Shoot Dry Weight

| **Origin** | **DF** | **Sum of Squares** | **Mean Square** | **F Value** | **Pr > F** |
| --- | --- | --- | --- | --- | --- |
| **Model** | 4 | 0.00002532 | 0.00000633 | 5.59 | 0.0125 |
| **Error** | 10 | 0.00001131 | 0.00000113 |  |  |
| **Total corr** | 14 | 0.00003663 |  |  |  |

| **R-Square** | **Var Coef.** | **MSE Root** | **Mean** |
| --- | --- | --- | --- |
| 0.691140 | 14.42552 | 0.001064 | 0.007373 |

| **Origin** | **DF** | **Anova SS** | **Mean Square** | **F Value** | **Pr > F** |
| --- | --- | --- | --- | --- | --- |
| **Ti** | 4 | 0.00002532 | 0.00000633 | 5.59 | 0.0125 |

| SSAS |
| --- |

ANOVA

Dependent variable: Leaf Fresh Weight

| **Origin** | **DF** | **Sum of Squares** | **Mean Square** | **F Value** | **Pr > F** |
| --- | --- | --- | --- | --- | --- |
| **Model** | 4 | 0.00400777 | 0.00100194 | 2.30 | 0.1304 |
| **Error** | 10 | 0.00436007 | 0.00043601 |  |  |
| **Total corr** | 14 | 0.00836784 |  |  |  |

| **R-Square** | **Var Coef.** | **MSE Root** | **Mean** |
| --- | --- | --- | --- |
| 0.478949 | 11.32978 | 0.020881 | 0.184300 |

| **Origin** | **DF** | **Anova SS** | **Mean Square** | **F Value** | **Pr > F** |
| --- | --- | --- | --- | --- | --- |
| **Ti** | 4 | 0.00400777 | 0.00100194 | 2.30 | 0.1304 |

| SAS |
| --- |

ANOVA

Dependent variable: Leaf Dry Weight

| **Origin** | **DF** | **Sum of Squares** | **Mean Square** | **F Value** | **Pr > F** |
| --- | --- | --- | --- | --- | --- |
| **Model** | 4 | 0.00003364 | 0.00000841 | 2.35 | 0.1248 |
| **Error** | 10 | 0.00003583 | 0.00000358 |  |  |
| **Total corr** | 14 | 0.00006947 |  |  |  |

| **R-Square** | **Var Coef.** | **MSE Root** | **Mean** |
| --- | --- | --- | --- |
| 0.484281 | 13.92442 | 0.001893 | 0.013593 |

| **Origin** | **DF** | **Anova SS** | **Mean Square** | **F Value** | **Pr > F** |
| --- | --- | --- | --- | --- | --- |
| **Ti** | 4 | 0.00003364 | 0.00000841 | 2.35 | 0.1248 |

| SAS |
| --- |

ANOVA

Dependent variable: Total Fresh Weight

| **Origin** | **DF** | **Sum of Squares** | **Mean Square** | **F Value** | **Pr > F** |
| --- | --- | --- | --- | --- | --- |
| **Model** | 4 | 0.04910590 | 0.01227648 | 2.21 | 0.1410 |
| **Error** | 10 | 0.05554577 | 0.00555458 |  |  |
| **Total corr** | 14 | 0.10465168 |  |  |  |

| **R-Square** | **Var Coef.** | **MSE Root** | **Mean** |
| --- | --- | --- | --- |
| 0.469232 | 13.71128 | 0.074529 | 0.543560 |

| **Origin** | **DF** | **Anova SS** | **Mean Square** | **F Value** | **Pr > F** |
| --- | --- | --- | --- | --- | --- |
| **Ti** | 4 | 0.04910590 | 0.01227648 | 2.21 | 0.1410 |

| SAS |
| --- |

ANOVA

Dependent variable: Total Dry Weight

| **Origin** | **DF** | **Sum of Squares** | **Mean Square** | **F Value** | **Pr > F** |
| --- | --- | --- | --- | --- | --- |
| **Model** | 4 | 0.00014923 | 0.00003731 | 1.60 | 0.2498 |
| **Error** | 10 | 0.00023380 | 0.00002338 |  |  |
| **Total corr** | 14 | 0.00038303 |  |  |  |

| **R-Square** | **Var Coef.** | **MSE Root** | **Mean** |
| --- | --- | --- | --- |
| 0.389603 | 14.47402 | 0.004835 | 0.033407 |

| **Origin** | **DF** | **Anova SS** | **Mean Square** | **F Value** | **Pr > F** |
| --- | --- | --- | --- | --- | --- |
| **Ti** | 4 | 0.00014923 | 0.00003731 | 1.60 | 0.2498 |

| SAS |
| --- |

ANOVA

t Tests (LSD) for Root Fresh Weight

| **Alpha** | 0.05 |
| --- | --- |
| **DF** | 10 |
| **Error of Mean Square** | 0.001951 |
| **t critical Value** | 2.22814 |
| **Least significant difference** | 0.0804 |

| **t Groups** | | **Mean** | **N** | **Ti** |
| --- | --- | --- | --- | --- |
|  | A | 0.23517 | 3 | 208.8 |
|  | A |  |  |  |
| B | A | 0.20123 | 3 | 52.2 |
| B | A |  |  |  |
| B | A | 0.19760 | 3 | 156.6 |
| B | A |  |  |  |
| B | A | 0.16757 | 3 | 104.4 |
| B |  |  |  |  |
| B |  | 0.15243 | 3 | 0 |

| Sistema SAS |
| --- |

ANOVA

t Tests (LSD) Root Dry Weight

| Note: | This test controls the Type I comparisonwise error rate, not the experimentwise error rate. |
| --- | --- |

| **Alpha** | 0.05 |
| --- | --- |
| **DF** | 10 |
| **Error of Mean Square** | 7.573E-6 |
| **t critical Value** | 2.22814 |
| **Least significant difference** | 0.005 |

| **Means with the same letter do not have significant differences.** | | | |
| --- | --- | --- | --- |
| **t Groups** | **Mean** | **N** | **Ti** |
| A | 0.013700 | 3 | 208.8 |
| A |  |  |  |
| A | 0.013633 | 3 | 52.2 |
| A |  |  |  |
| A | 0.012033 | 3 | 0 |
| A |  |  |  |
| A | 0.011667 | 3 | 156.6 |
| A |  |  |  |
| A | 0.011167 | 3 | 104.4 |

| SAS |
| --- |

ANOVA

t Tests (LSD) for Shoot Fresh Weight

| Note: | This test controls the Type I comparisonwise error rate, not the experimentwise error rate. |
| --- | --- |

| **Alpha** | 0.05 |
| --- | --- |
| **DF** | 10 |
| **Error of Mean Square** | 0.000267 |
| **t critical Value** | 2.22814 |
| **Diferencia menos significativa** | 0.0297 |

| **Means with the same letter do not have significant differences.** | | | | |
| --- | --- | --- | --- | --- |
| **t Groups** | | **Mean** | **N** | **Ti** |
|  | A | 0.18843 | 3 | 0 |
|  | A |  |  |  |
|  | A | 0.18727 | 3 | 208.8 |
|  | A |  |  |  |
| B | A | 0.18147 | 3 | 156.6 |
| B |  |  |  |  |
| B | C | 0.15583 | 3 | 52.2 |
|  | C |  |  |  |
|  | C | 0.12930 | 3 | 104.4 |

| SAS |
| --- |

ANOVA

t Tests (LSD) for Shoot Dry Weight

| Note: | This test controls the Type I comparisonwise error rate, not the experimentwise error rate. |
| --- | --- |

| **Alpha** | 0.05 |
| --- | --- |
| **DF** | 10 |
| **Error of Mean Square** | 1.131E-6 |
| **t critical Value** | 2.22814 |
| **Least significant difference** | 0.0019 |

| **Means with the same letter do not have significant differences.** | | | |
| --- | --- | --- | --- |
| **t Groups** | **Mean** | **N** | **Ti** |
| A | 0.0088333 | 3 | 0 |
| A |  |  |  |
| A | 0.0080667 | 3 | 208.8 |
| A |  |  |  |
| A | 0.0075333 | 3 | 52.2 |
| A |  |  |  |
| A | 0.0074667 | 3 | 156.6 |
|  |  |  |  |
| B | 0.0049667 | 3 | 104.4 |

| SAS |
| --- |

ANOVA

t Tests (LSD) for Leaf Fresh Weight

| Note: | This test controls the Type I comparisonwise error rate, not the experimentwise error rate. |
| --- | --- |

| **Alpha** | 0.05 |
| --- | --- |
| **DF** | 10 |
| **Error of Mean Square** | 0.000436 |
| **t critical Value** | 2.22814 |
| **Least significant difference** | 0.038 |

| **Means with the same letter do not have significant differences.** | | | | |
| --- | --- | --- | --- | --- |
| **t Groups** | | **Mean** | **N** | **Ti** |
|  | A | 0.21203 | 3 | 208.8 |
|  | A |  |  |  |
| B | A | 0.18493 | 3 | 156.6 |
| B | A |  |  |  |
| B | A | 0.18483 | 3 | 0 |
| B | A |  |  |  |
| B | A | 0.17847 | 3 | 52.2 |
| B |  |  |  |  |
| B |  | 0.16123 | 3 | 104.4 |

| SAS |
| --- |

ANOVA

t Tests (LSD) para Leaf Dry Weight

| Note: | This test controls the Type I comparisonwise error rate, not the experimentwise error rate. |
| --- | --- |

| **Alpha** | 0.05 |
| --- | --- |
| **DF** | 10 |
| **Error of Mean Square** | 3.583E-6 |
| **t critical Value** | 2.22814 |
| **Least significant difference** | 0.0034 |

| **Means with the same letter do not have significant differences.** | | | | |
| --- | --- | --- | --- | --- |
| **t Groups** | | **Mean** | **N** | **Ti** |
|  | A | 0.015467 | 3 | 0 |
|  | A |  |  |  |
| B | A | 0.014533 | 3 | 52.2 |
| B | A |  |  |  |
| B | A | 0.014200 | 3 | 156.6 |
| B | A |  |  |  |
| B | A | 0.012433 | 3 | 208.8 |
| B |  |  |  |  |
| B |  | 0.011333 | 3 | 104.4 |

| SAS |
| --- |

ANOVA

t Tests (LSD) for Total Fresh Weight

| Note: | This test controls the Type I comparisonwise error rate, not the experimentwise error rate. |
| --- | --- |

| **Alpha** | 0.05 |
| --- | --- |
| **DF** | 10 |
| **Error of Mean Square** | 0.005555 |
| **t critical Value** | 2.22814 |
| **Least significant difference** | 0.1356 |

| **Means with the same letter do not have significant differences.** | | | | |
| --- | --- | --- | --- | --- |
| **t Groups** | | **Mean** | **N** | **Ti** |
|  | A | 0.63447 | 3 | 208.8 |
|  | A |  |  |  |
| B | A | 0.56400 | 3 | 156.6 |
| B | A |  |  |  |
| B | A | 0.53553 | 3 | 52.2 |
| B | A |  |  |  |
| B | A | 0.52570 | 3 | 0 |
| B |  |  |  |  |
| B |  | 0.45810 | 3 | 104.4 |

| SAS |
| --- |

ANOVA

t Tests (LSD) for Total Dry Weight

| **Alpha** | 0.05 |
| --- | --- |
| **DF** | 10 |
| **Error of Mean Square** | 0.000023 |
| **t critical Value** | 2.22814 |
| **Least significant difference** | 0.0088 |

|  | | | | |
| --- | --- | --- | --- | --- |
| **Means with the same letter do not have significant differences.** | | | | |
| **t Groups** | | **Mean** | **N** | **Ti** |
|  | A | 0.036333 | 3 | 0 |
| B | A | 0.035700 | 3 | 52.2 |
| B | A | 0.034200 | 3 | 208.8 |
| B | A | 0.033333 | 3 | 156.6 |
| B |  | 0.027467 | 3 | 104.4 |

| SAS |
| --- |

ANOVA

| **Ti** | **N** | **Root Fresh Weight** | | **Root Dry Weight** | | **Shoot Fresh Weight** | | **Shoot Dry Weight** | | **Leaf Fresh Weight** | | **Leaf Dry Weight** | | **Total Fresh Weight** | | **Total Dy Weight** | |
| --- | --- | --- | --- | --- | --- | --- | --- | --- | --- | --- | --- | --- | --- | --- | --- | --- | --- |
|  |  | **Mean** | **SD** | **Mean** | **SD** | **Mean** | **SD** | **Mean** | **SD** | **Mean** | **SD** | **Mean** | **SD** | **Mean** | **SD** | **Mean** | **SD** |
| **0** | **3** | 0.15243333 | 0.06005117 | 0.01203333 | 0.00260064 | 0.18843333 | 0.01340050 | 0.00883333 | 0.00096090 | 0.18483333 | 0.01600323 | 0.01546667 | 0.00176163 | 0.52570000 | 0.08869927 | 0.03633333 | 0.00501232 |
| **52.2** | **3** | 0.20123333 | 0.01721753 | 0.01363333 | 0.00133167 | 0.15583333 | 0.01554295 | 0.00753333 | 0.00085049 | 0.17846667 | 0.02685151 | 0.01453333 | 0.00166533 | 0.53553333 | 0.05929859 | 0.03570000 | 0.00355949 |
| **104.4** | **3** | 0.16756667 | 0.01352122 | 0.01116667 | 0.00360185 | 0.12930000 | 0.01686090 | 0.00496667 | 0.00060277 | 0.16123333 | 0.01306917 | 0.01133333 | 0.00115902 | 0.45810000 | 0.03734983 | 0.02746667 | 0.00508167 |
| **156.6** | **3** | 0.19760000 | 0.04090440 | 0.01166667 | 0.00270617 | 0.18146667 | 0.00184481 | 0.00746667 | 0.00085049 | 0.18493333 | 0.01838813 | 0.01420000 | 0.00204206 | 0.56400000 | 0.05917711 | 0.03333333 | 0.00545558 |
| **208.8** | **3** | 0.23516667 | 0.06320454 | 0.01370000 | 0.00300500 | 0.18726667 | 0.02502006 | 0.00806667 | 0.00170978 | 0.21203333 | 0.02634394 | 0.01243333 | 0.00255408 | 0.63446667 | 0.10720104 | 0.03420000 | 0.00484974 |
