# Supplementary material for: Biostimulant effects of titanium dioxide nanoparticles on germination and initial growth of tomato: evidence of hormesis
Source: PeerJ. 2025 Dec 16;13:e20516. doi: 10.7717/peerj.20516 (PMC12716138; doi:10.7717/peerj.20516)
Supplement: Supplemental Information 6 [file peerj-13-20516-s006.docx]

**Re: Manuscript #114472**

**Previous title:** *Titanium enhances germination, fresh biomass accumulation and initial growth in tomato and stimulates stem and root length in a hormetic manner*

**Revised title:** *Biostimulant effects of titanium dioxide nanoparticles on germination and initial growth of tomato: Evidence of hormesis*

We respectfully submit the revised version of our manuscript, incorporating the requested changes from the first round of peer review. In this letter, we would like to explain the inclusion of a new co-author and confirm the completeness of the current submission.

1. **Addition of Dr. Jorge Luis Mejía-Méndez as Co-Author**

We have added Dr. Jorge Luis Mejía-Méndez to the list of authors, as he has made substantial contributions to the revision of the manuscript. All previous authors have reviewed and approved this addition. The rationale for his inclusion is as follows:

a) During the first round of peer review, the reviewers requested that we include zeta potential values for the nanoparticles used in our study.
b) As corresponding author, I reached out to Dr. Mejía-Méndez to inquire whether he could assist with these measurements.
c) He kindly agreed and conducted a full physicochemical characterization of the samples, including determination of hydrodynamic diameter, surface charge, and polydispersity index. He subsequently calculated the zeta potential values for all nanoparticle treatments.
d) In addition, he designed the graphs presented in Figure 1, contributed directly to the Results and significantly enhanced the Discussion section of the revised manuscript.
e) He thoroughly reviewed the entire manuscript and made substantial contributions to improving the final version we are now submitting.

Please note that Dr. Mejía-Méndez was not included in the original submission, as zeta potential measurements were not initially planned. This component was incorporated solely in response to reviewer requests. All co-authors have confirmed their agreement with this change via the confirmation email system.

2. **Documentation of Contributions**

Dr. Mejía-Méndez’s contributions have been clearly marked in the revised manuscript using the track changes and commenting functionalities. Each relevant section has been annotated to reflect his input.

3. **Final Submission Materials**

We confirm that the clean version of the manuscript and the tracked changes version are now fully aligned. Both reflect all requested revisions, including the addition of Dr. Mejía-Méndez and his respective contributions, in response to the editorial decision issued on April 28, 2025.

Should you require any additional information or clarification, please do not hesitate to contact me.

Sincerely,

**Dr. Fernando C. Gómez-Merino**

Corresponding Author

On behalf of all co-authors
